# Impact of the COVID-19 pandemic on hepatitis C care across the cascade of care: a scoping review

**DOI:** 10.1186/s12879-026-13799-1

**Published:** 2026-06-13

**Authors:** Angelo Lamadrid, Ignacio Leiva-Escobar, Alejandro Soza, Wilm Quentin

**Affiliations:** 1 Department of Health Care Management, Technische Universität Berlin, Berlin, Germany; 2 Institute of Health Services Research in Dentistry, University of Münster, Münster, Germany; 3 Department of Gastroenterology, School of Medicine, Pontificia Universidad Católica de Chile, Santiago, Chile; 4 Chair of Planetary & Public Health, University of Bayreuth, Bayreuth, Germany; 5 German West-African Centre for Global Health and Pandemic Prevention, Kwame Nkrumah University of Science and Technology (KNUST), Kumasi, Ghana

**Keywords:** Hepatitis C, Continuum of care, COVID-19, Cascade of care

## Abstract

**Background:**

The COVID-19 pandemic was associated with widespread health services disruption, including challenges in the management of chronic infectious diseases. However, evidence regarding reported changes in hepatitis C virus (HCV) care during this period remains limited and fragmented. We aimed to map the available evidence on reported changes in HCV care during the COVID-19 pandemic across the HCV cascade of care.

**Method:**

We conducted a scoping review following the Arksey and O’Malley methodological framework. Searches of Medline (PubMed), Cochrane Library, Embase, Scopus, Web of Science, and grey literature identified studies reporting pandemic-related changes in HCV diagnosis, linkage to care, treatment, or cure from; March 2020 to March 2025. Data were extracted on the study setting, stage of the cascade of care, and observed changes in HCV care, and synthesized narratively.

**Results:**

Twenty-eight studies met the inclusion criteria. Eighteen were conducted in Europe and North America. Most studies found declines in HCV care across all stages of the cascade of care: diagnosis (15 of 21 studies), linkage to care (9 of 10), treatment initiation or completion (18 of 27), and cure (6 of 13). Among the included studies, linkage to care was the stage most consistently reported as disrupted.

**Conclusion:**

Evidence on reported changes in the HCV cascade of care during the COVID-19 pandemic is limited and geographically skewed toward high-income countries. Available data suggest service reductions across all stages, with linkage to care being the stage most frequently reported as disrupted among the studies that assessed it. Strengthening surveillance, ensuring routine monitoring at all stages of the cascade of care, and building resilient HCV services are critical to safeguarding progress toward the 2030 hepatitis elimination goal.

**Supplementary Information:**

The online version contains supplementary material available at 10.1186/s12879-026-13799-1.

## Background

Eliminating viral hepatitis as a public health problem by 2030 is part of the Sustainable Development Goals [1]. Additionally, the World Health Organization (WHO) has proposed, as a specific impact target for hepatitis C virus (HCV), to reduce HCV mortality by 65% and new cases of chronic HCV by 80% [2, 3]. Ten core indicators were developed to provide countries with a standardized framework for monitoring and evaluating their health system response toward HCV elimination by 2030. This included the cascade of care framework [4].

The cascade of care is a conceptual model that illustrates how individuals within a population progress through various stages required to manage a disease [3]. Initially, it was used to assess the effectiveness and performance of the healthcare systems at different points in the HIV care continuum [3]. The Joint United Nations Programme on HIV/AIDS (UNAIDS) used this framework to develop the 95-95-95 strategy, which aims to diagnose at least 95% of all people living with HIV, treat at least 95% of all people diagnosed, and achieve viral suppression in at least 95% of people receiving antiretroviral treatment by 2025 [5].

Similarly, the HCV cascade of care comprises diagnosis, treatment, and cure [3, 4]. Previous studies have suggested additional stages, such as linkage to care (i.e., referral to specialized medical care after diagnosis) [6], could be included, but this will depend on data availability and the country’s priorities [3].

COVID-19-related health service disruptions have had detrimental consequences on managing HCV in several countries [7–9]. In fact, according to a modeling study conducted for 110 countries, a one-year delay in HCV screening, diagnosis, and treatment could result in 44,800 excess cases of hepatocellular carcinoma and 72,300 excess deaths from liver disease [10].

The cascade of care is not only a surveillance system for monitoring progress toward HCV elimination. It can also be used to determine the impact of COVID-19 on HCV care [11]. This framework is particularly useful because it conceptualizes HCV care as a continuum, rather than as a set of isolated service indicators or clinical outcomes [6].

To the best of our knowledge, existing reviews on the relationship between COVID-19 and HCV have not focused on the entire cascade of care but have instead focused on individual clinical outcomes [12, 13] or selected services [14]. To fill this gap, we aimed to map the available evidence on reported changes in HCV care during the COVID-19 pandemic across the HCV cascade of care. More specific objectives included: (1) to describe the characteristics of studies, (2) to identify the stages of the HCV cascade of care addressed by each study, and (3) to summarize observed changes in HCV care at different stages of the cascade.

## Methods

We conducted this scoping review in line with guidance from the Joanna Briggs Institute [15] and the Arksey and O’Malley methodological framework [16]. Additionally, we report our findings according to the Preferred Reporting Items for Systematic Reviews and Meta-Analysis extension for Scoping Reviews (PRISMA-ScR) [17]. The protocol was registered at OSFhome [18].

### Eligibility criteria

We defined the eligibility criteria based on the PCC (population, concept, context) framework [15]:

P: People diagnosed with hepatitis C, healthcare providers or managers of hepatitis C care, or members of civil society working in hepatitis C. Studies merging people diagnosed with HCV with other viral hepatitis, sexually transmitted infections, or other blood-borne diseases were excluded.

C (concept): assessment of at least two stages of the hepatitis C cascade of care, which is aligned with previous studies using cascade of care framework [19–21]. The stages were defined as follows: (i) diagnosis referred to studies reporting the number or proportion of people who were tested for hepatitis C using anti-HCV or HCV RNA testing [4] as well as the number of anti-HCV or HCV RNA tests performed; (ii) linkage to care referred to studies reporting the number or proportion of people diagnosed with hepatitis C who are receiving specialist evaluation (e.g. medical evaluation, liver assessment or virological biomarker testing) [4]; (iii) treatment referred to studies reporting the number or proportion of people diagnosed with chronic hepatitis C who started or completed treatment [4] and, (iv) cure referred to studies reporting the number or proportion of people diagnosed with chronic hepatitis C who received treatment and had a sustained virologic response (SVR) at least 12 weeks after completion of treatment [4].

Studies focusing exclusively on strategies implemented to mitigate the impact of COVID-19 were excluded if they did not report pre-pandemic data or did not allow assessment of change in the HCV cascade of care.

C (context): the COVID-19 pandemic caused by the SARS-CoV-2 virus. Studies that used only data collected before March 2020, when the WHO declared COVID-19 a pandemic, were excluded. Pre-pandemic periods were defined pragmatically as any comparison period reported prior to March 2020. We did not standardize comparator periods across studies, in keeping with the exploratory nature of the scoping review.

Additionally, we included quantitative and qualitative studies. Commentaries, opinion pieces, editorials, and conference abstracts were also included as long as they addressed our research question. We excluded secondary studies (systematic reviews, scoping reviews, literature reviews), modeled data, protocols, or guidelines. However, in those relevant to our research question, we searched their reference list for additional studies that met the eligibility criteria. There was no restriction on languages. Studies written in languages other than English or Spanish were translated using Google Translate for screening and data extraction.

### Search strategy and selection process

The search strategy combined synonyms and associated terms related to (1) hepatitis C, (2) cascade of care, and (3) COVID-19, using Medical Subject Headings (MeSH) and the Boolean operators “AND” or “OR”. An experienced librarian at the Technische Universität Berlin checked the search strategy. A detailed description of our search strategy is available in Additional file 1.

On 16 December 2024, one of the authors (AL) searched Medline (PubMed), the Cochrane Library database, Embase, Scopus, and Web of Science. The same author updated the searches on 15 March 2025. Additionally, AL conducted a grey literature search on 18 December 2024 and 17 March 2025 by manually reviewing the websites of relevant international organizations, medical societies, and non-governmental organizations working in hepatitis C, including the WHO (https://www.who.int), the European Association for the Study of the Liver (https://easl.eu), the American Association for the Study of Liver Diseases (https://www.aasld.org), the CDA Foundation (https://cdafound.org), and the World Hepatitis Alliance (https://www.worldhepatitisalliance.org) [14]. Grey literature sources were screened using the same eligibility criteria already mentioned.

All retrieved records were exported to EndNote, and duplicates were removed.

Two authors (AL and ILE) independently screened and identified studies in two stages. First, titles and abstracts were screened against eligibility criteria. Second, the pre-selected studies were assessed for inclusion based on the full texts. Disagreements at either stage were resolved through discussion and consensus.

### Data extraction

The selected studies were exported to Microsoft Excel for analysis. We used the data charting form proposed by Arksey and O’Malley [16] (comprising author, year of publication, country where the research was conducted, aims, methodology, outcomes, and results). To respond to our research question, we focused on i*)* the region (and country) where the study was conducted, (ii) the outcome studied (stage of HCV cascade of care involved), and (iii) the results of the impact of the COVID-19 pandemic on hepatitis C care based on the cascade of care framework.

When the information was unavailable, we wrote to the corresponding author to confirm the missing data. Data extraction was conducted by two authors (AL and ILE). As in the previous section, any disagreement was resolved by consensus.

### Collating, summarizing, and reporting the results

We used summary tables to organize studies according to author and year of publication, study location (country and continent), study design, reported outcomes, and main results.

Given the substantial heterogeneity across study designs, populations, settings, outcome definitions, and comparison periods, we used a categorical narrative synthesis to summarize the direction of findings within each stage of the HCV cascade of care. Reported results were classified into four categories: (i) decrease, defined as a numerical or percentage reduction compared with the pre-COVID-19 period; (ii) increase, defined as a numerical or percentage rise compared with the pre-COVID-19 period; (iii) no change, defined as no difference compared with the pre-COVID-19 period; and (iv) mix, defined as studies reporting findings in more than one direction during the COVID-19 period.

When statistical significance was reported, findings with p-values ≥ 0.05 were classified as no change, regardless of the numerical direction of the estimate. When a study reported multiple indicators within the same cascade stage, we classified the findings according to their overall direction. If all indicators were consistent, the study was assigned a single category; if indicators within the same stage differed in direction, the finding was classified as mixed. However, when studies reported an initial decline followed by a rebound that remained below the pre-COVID-19 levels, the finding was classified as a decrease rather than mixed.

For surveys, we classified the results based on the response category reported by the highest proportion of participants.

This approach was chosen to synthesize across a broad, methodologically diverse evidence base, in keeping with the purpose of a scoping review, which does not formally assess study quality or generalizability [16].

## Results

A total of 5283 sources were identified, including 5275 records through searches of Medline (PubMed), the Cochrane Library, Embase, Scopus, and Web of Science, along with 8 additional sources identified through other methods. Of the 5275 records identified through database searching, 738 duplicates and 858 records published before the declaration of the COVID-19 pandemic were removed, leaving 3679 records for title and abstract screening. Of these, 3438 records were excluded, and 3 additional records were excluded because the abstracts were inaccessible. Therefore, 238 reports were assessed for full-text eligibility.

The 8 additional sources identified through other methods were assessed separately, but all were excluded.

At the full-text review stage, 210 reports were excluded, primarily due to wrong population (*n* = 6), wrong concept (*n* = 138), wrong context (*n* = 27), and wrong study type (*n* = 39).

**Fig. 1:**
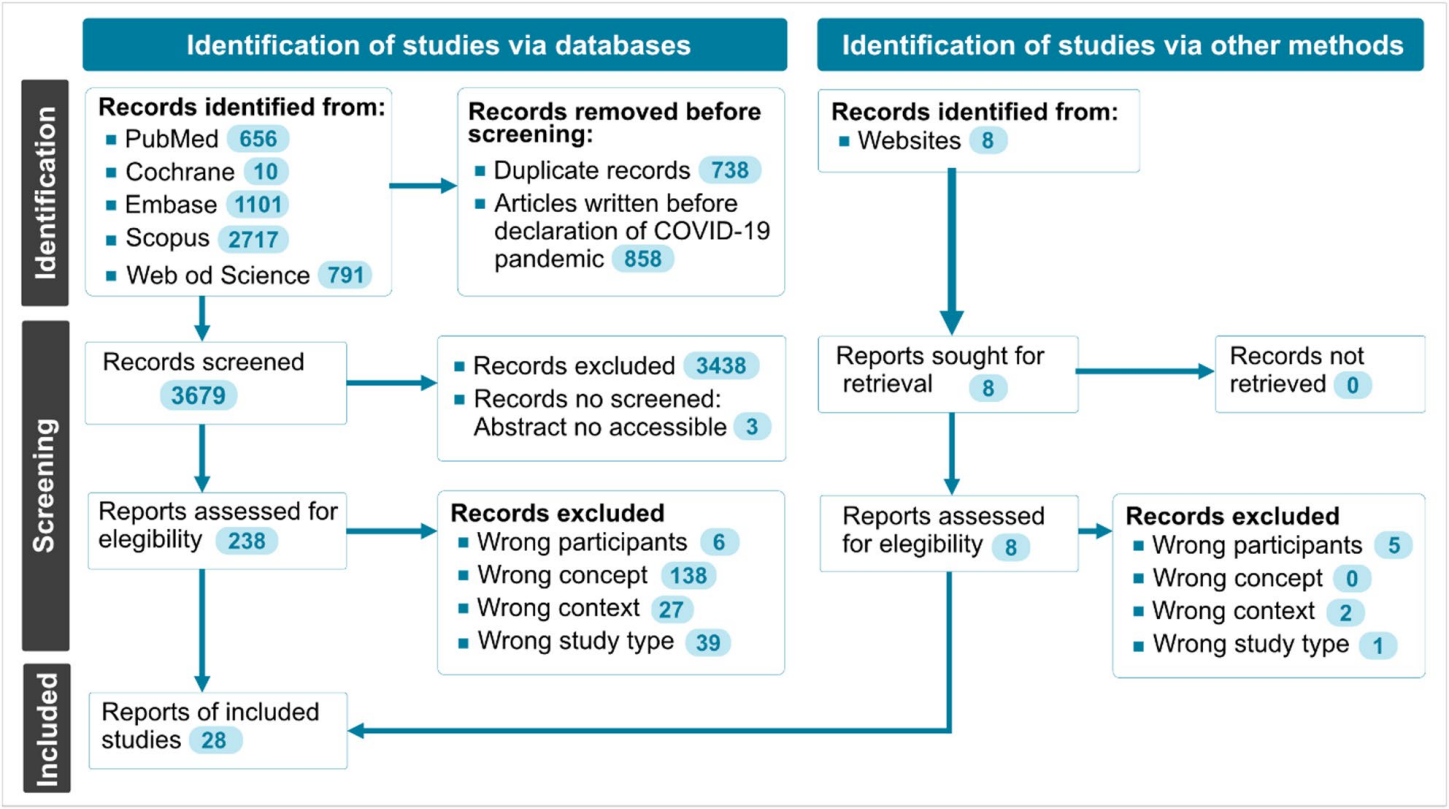
PRISMA scoping review flow chart

A total of 28 studies were included in the review (Fig. 1). However, not all studies contributed data for every stage of the HCV cascade of care. Therefore, the number of studies included in the denominator varied across stages. A list of the included studies is provided in Additional file 2.

### Characteristics of included studies

Table 1 summarizes the characteristics of included studies. Eleven studies were published in 2022 [11, 22–31] and six studies in 2023 [32–37]. Twenty-three were peer-reviewed articles [11, 22–30, 32–34, 38–47], and five were conference abstracts/presentations [31, 35–37, 48]. Twenty-five studies were quantitative [11, 22–28, 30–34, 36–38, 40–48], mostly observational and retrospective data analyses (*n* = 9 each). Twenty-four studies included people diagnosed with hepatitis C [11, 22, 23, 25, 26, 28–33, 35–39, 41–48]. Fifteen studies assessed the impact of COVID-19 on two stages of the HCV cascade of care [11, 25, 26, 28, 34, 36–43, 45, 46], while 13 assessed three or more stages [22–24, 27, 29–33, 35, 44, 47, 48].

**Table 1:** Characteristics of included studies (*n* = 28)

Characteristics	Frequency (%)
**Year of publication**	
2021	5 (17.9)
2022	11 (39.3)
2023	6 (21.4)
2024	4 (14.3)
2025	2 (7.1)
**Type of Study**	
Peer-reviewed articles	23 (82.1)
Conference abstracts/presentations	5 (17.9)
**Study design**	
Quantitative	25 (89.2)
Quasi-experimental	1 (3.6)
Report	1 (3.6)
Modelling study*	1(3.6)
**Quantitative study subtype** (***n*** = 25) **	
Survey	4 (16)
Observational	9 (36)
Retrospective	9 (36)
Cohort/Longitudinal	2 (8)
Ecological	1 (4)
**Participants**	
People diagnosed with hepatitis C	24 (85.7)
Health providers or managers	4 (14.3)
**Number of HCV cascade of care stages studied**	
2	15 (53.6)
3	11 (39.3)
4	2 (7.1)

*One modelling study was included because, although its main objective was modelling, it reported descriptive information on observed changes in the HCV cascade of care during the COVID-19 pandemic that addressed the research question

** Quantitative study subtypes are presented as a secondary classification within studies identified as quantitative (*n* = 25). Categories were based on how study designs were reported in the original articles

### Regions and HCV stages of the HCV cascade of care most studied

**Fig. 2:**
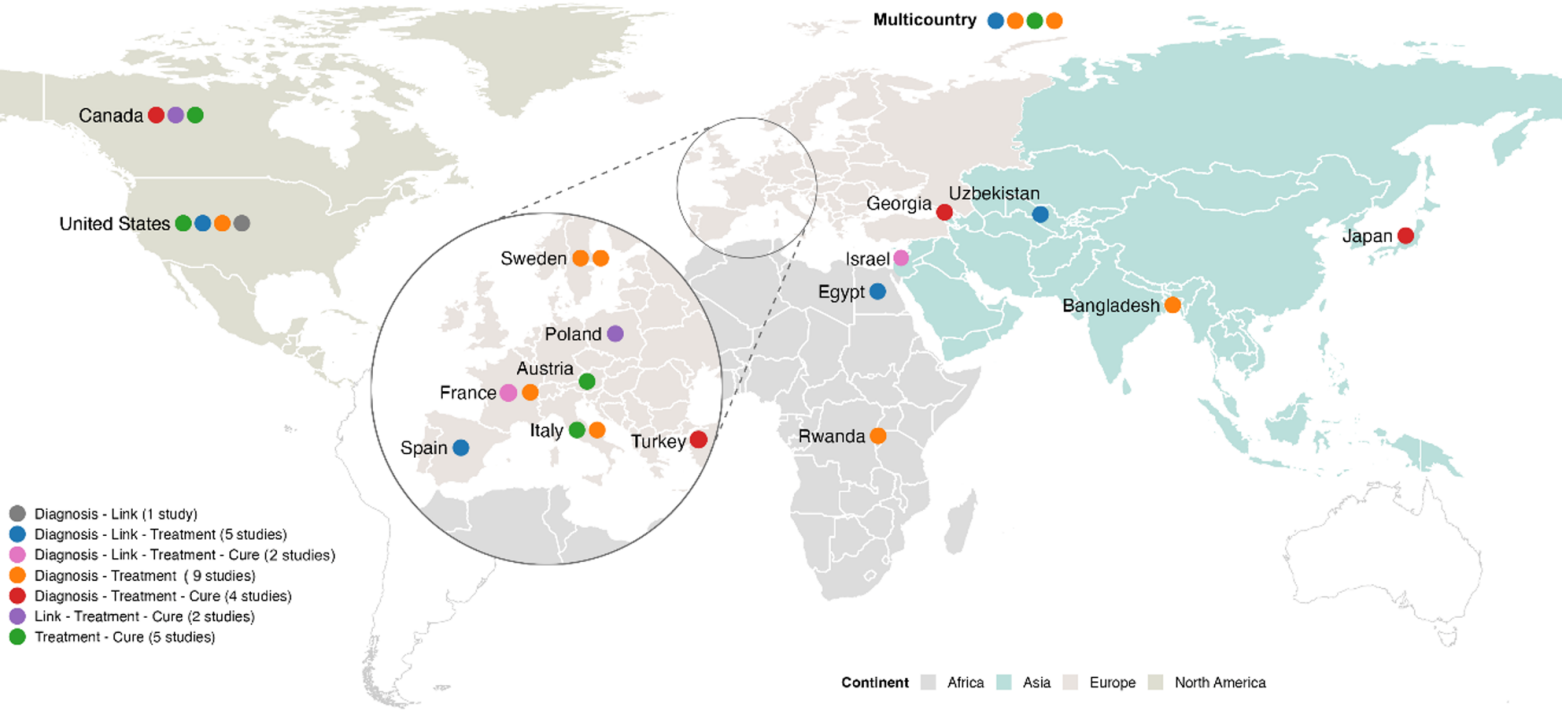
Geographic distribution of studies

Eighteen studies were conducted in only two regions: Eleven in Europe [22, 23, 25, 30, 32, 38, 39, 41–43, 48], and seven in North America [28, 29, 31, 36, 44–46]. The remaining studies originated from Africa (*n* = 2) [26, 35], Asia (*n* = 4) [24, 33, 34, 47], or included participants from multiple regions (*n* = 4) [11, 27, 37, 40] (Fig. 2).

Across the 28 included studies, the most frequently assessed stages of the cascade of care were diagnosis (*n* = 21 studies) and treatment (*n* = 27 studies), followed by cure (*n* = 13 studies) and linkage to care (*n* = 10 studies).

Diagnosis and treatment were the most evaluated stages in combination. Nine studies focused exclusively on these two stages [26, 34, 37–41, 43, 45], while an additional 11 studies assessed them alongside at least one other stage [23, 24, 27, 29, 30, 32, 33, 35, 44, 47, 48].

### Impact of the COVID-19 pandemic on HCV cascade of care

#### Diagnosis

**Fig. 3:**
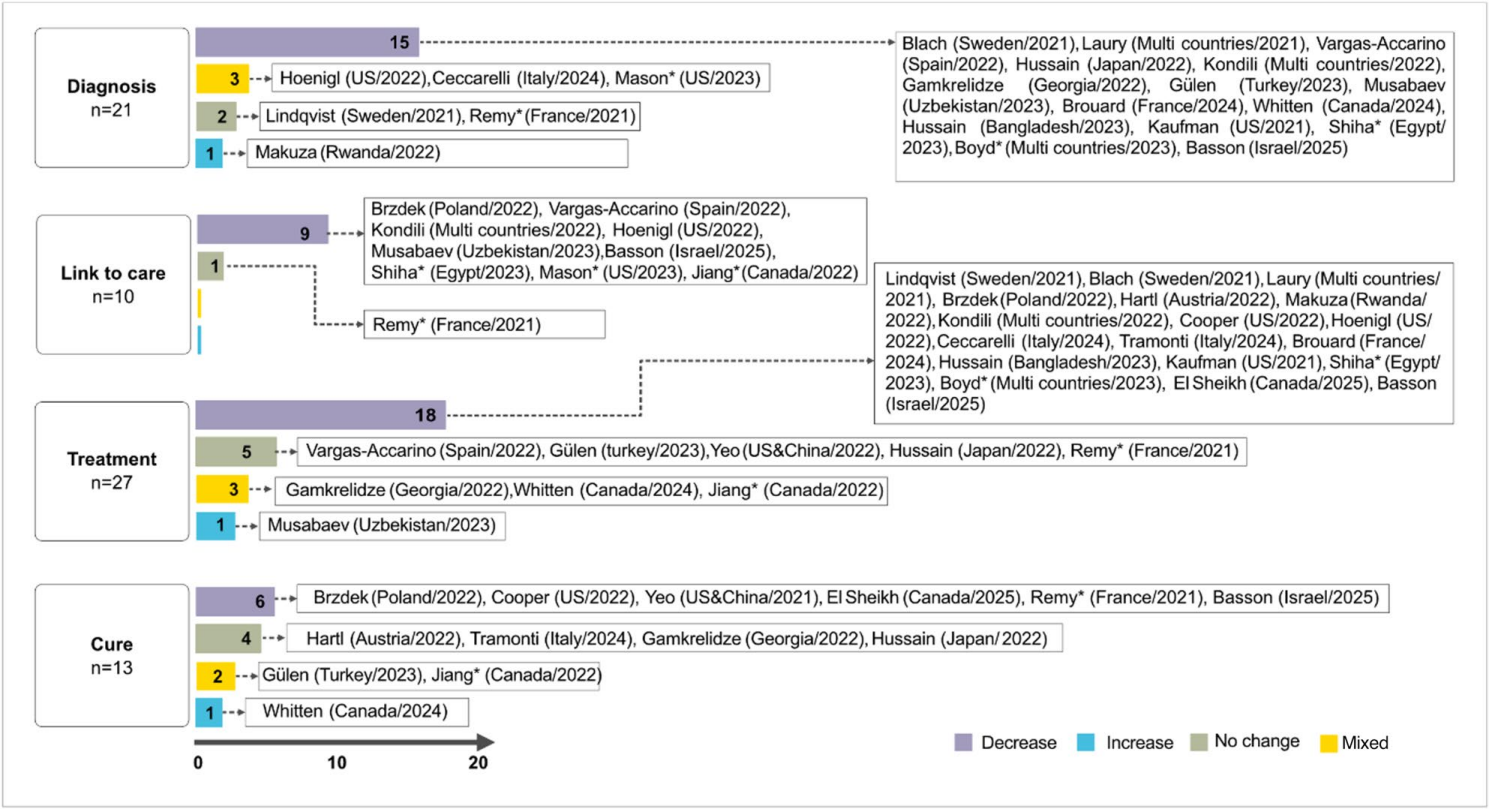
Relative impact of COVID-19 pandemic on HCV cascade of care. *Abstract/conference presentation

Of the 21 studies that examined HCV diagnosis, 15 reported a decrease in anti-HCV or HCV RNA testing [23, 24, 27, 30, 32–35, 37, 39, 40, 43–45, 47] (Fig. 3). The largest reported decline was in Egypt, where HCV RNA test requests fell by 60.7% between 2019 and 2020, resulting in an 86.9% drop in diagnoses [35](abstract/conference presentation).

Two studies, from Sweden [38] and France [48], reported no decrease in testing among injection drug users and prisoners, respectively. Three studies reported mixed effects, such as an initial decline followed by recovery to pre-pandemic levels [29, 41], or an increase in anti-HCV testing alongside a decrease in HCV RNA testing [36]. One study from Rwanda reported an overall increase in both anti-HCV and HCV RNA testing following the start of the pandemic [26].

#### Linkage to care

Ten studies assessed the effect on linkage to care. Of these, nine reported a reduction in the number of people diagnosed with hepatitis C linked to medical care [22, 23, 27, 29, 31, 33, 35, 36, 47]. One study from France reported no change in liver assessment between 2019 and 2020 among inmates with positive HCV RNA [48] (Abstract/conference presentation).

#### Treatment

Among the 27 studies evaluating treatment, 18 reported a decrease in treatment initiation or completion during the COVID-19 pandemic [22, 25–29, 34, 35, 37–43, 45–47]. Five studies reported no significant change in treatment initiation [23, 24, 48] or completion [11, 32].

Three studies described mixed effects. Two reported a decrease in the number of people treated but an increase in the percentage of treatment completion compared with the pre-pandemic period [30, 31]. Another study displayed a decline in treatment in 2020 compared to 2017, followed by a rebound in 2021 [44]. One study reported an increase in the number of people treated [33].

#### Cure

Thirteen studies assessed the effect on sustained virologic response. Six reported a decrease in SVR during the pandemic [11, 22, 28, 46–48]. Four studies reported no change [24, 25, 30, 42]. One reported an overall increase in SVR [44], and two studies reported mixed effects [31, 32].

A complementary summary table of results is provided in Additional file 3.

## Discussion

To the best of our knowledge, this is the first scoping review to assess the impact of the COVID-19 pandemic on HCV care using the cascade of care framework. We identified 28 studies assessing changes across diagnosis, linkage to care, treatment, and cure. Three main findings emerged. First, most research was conducted in Europe and North America, with relatively limited evidence from regions carrying the highest global HCV burden. Second, the literature focused mainly on diagnosis and treatment, leaving other stages of the cascade of care underexplored. Third, linkage to care, the critical step connecting diagnosis to treatment, was the stage most consistently reported as disrupted.

The identified concentration of studies in high-income countries mirrors patterns reported by previous systematic reviews on the impact of COVID-19 across different health outcomes [49, 50]. The Eastern Mediterranean and South-East Asia regions together account for more than 40% of the global HCV burden [51]. Yet, we found only two studies from these regions, one from Egypt [35], which was an abstract/conference presentation, and one from Bangladesh [34]. This discrepancy between research focus and disease burden exemplifies persistent global research inequities, which hamper our understanding of the global impact of the pandemic on HCV care. Resource constraints in low- and middle-income countries meant that funds had to be reallocated during the pandemic toward COVID-19 control measures [7, 52], and telemedicine was less available [53]. These factors may have contributed to greater disruptions of HCV care provision, which may not be captured in studies from high-income countries.

WHO recommends monitoring the number of people infected, diagnosed, treated, and cured [3]. However, most existing studies focus only on diagnosis and treatment. This focus is understandable, as diagnosis is the entry point to all subsequent care [4], and treatment with direct-acting antivirals achieves cure in over 95% of patients [54]. However, linkage to care emerged as the stage most frequently reported as disrupted among studies that assessed it. Even before the pandemic, many people diagnosed with HCV did not progress to specialist assessment [55]. Broader literature suggests that lockdowns, staff redeployment, and fear of infection may have contributed to longer waiting times and increased loss to follow-up [56]. These service-level barriers may also have interacted with individual and contextual factors -homelessness, substance use, stigma, poverty, and lack of health insurance- that already limited access to HCV care prior to the pandemic [57]. The pandemic’s socioeconomic consequences, including widespread job losses and increased poverty, may have further intensified these challenges [58]. Rising alcohol and drug use during this period, particularly among people with pre-existing mental-health vulnerabilities, may also have reduced engagement with care [59]. Although these factors were not systematically evaluated in the included studies, evidence from the broader literature may help contextualize the disruptions in HCV care.

In contrast, evidence from the additional literature suggests that HIV services may have been more resilient during the pandemic. Studies indicate that antiretroviral therapy provision was largely maintained, supported by measures such as community-based drug distribution [60] and strong collaboration with civil-society organizations [61]. This experience highlights how targeted mitigation strategies may help preserve continuity of care during health emergencies and may offer relevant lessons for strengthening HCV services.

Our findings have important implications for policymakers and researchers. First, the geographic concentration of existing studies underscores the need to strengthen surveillance and research capacities in regions with the highest disease burden. Expanding use of the cascade of care as a monitoring framework would help identify service disruptions early [4] and ensure a truly global evidence base for future crises.

Second, programs should broaden their focus beyond diagnosis and treatment. Routine reporting of all stages of the cascade of care would allow health systems to detect bottlenecks and plan interventions that support progress toward the 2030 hepatitis elimination goal [4].

Third, the frequent reporting of disruptions in linkage to care among the studies that assessed it may highlight the need for targeted measures to build system resilience. Strategies proven in HIV services, such as community-based testing, decentralized drug delivery, partnerships with civil society, and telemedicine, could be adapted for HCV to maintain follow-up when access to specialized care is limited. Addressing socioeconomic determinants such as poverty, unstable housing, and substance use must be integral to these efforts, as they directly affect patients’ ability to engage with care.

### Strengths and limitations

This review maps where evidence is available and summarizes the direction of reported changes across the HCV cascade of care. It does not assess the methodological quality of the included studies [16], nor does it estimate the magnitude of pandemic-related disruption. Therefore, the findings should be interpreted as an overview of reported patterns and evidence gaps, rather than as a measure of the reliability or size of the pandemic’s impact.

Furthermore, the heterogeneous designs of the included studies, including differences in populations, outcomes, and time windows used for pre-pandemic and pandemic comparisons, complicate evidence synthesis and limit our ability to draw firm conclusions about the pandemic’s impact. Nevertheless, identifying where evidence is concentrated, which stages were most frequently reported as disrupted, and how these findings may relate to broader socioeconomic context provide a useful basis for policy planning and for strengthening the resilience of HCV care systems in preparation for future public health emergencies.

Some explanatory mechanisms discussed in the Discussion were informed by broader literature rather than directly assessed in the included studies and should therefore be considered contextual interpretations rather than direct findings of this review.

Additionally, we acknowledge that our eligibility criterion excluded studies focusing on a single stage of the HCV cascade of care, potentially limiting the capture of all available evidence on stage-specific impacts. However, we prioritized studies focused on assessing the continuum of care rather than isolated clinical outcomes. Likewise, our categorization of findings according to direction of change may have simplified heterogeneous indicators within the same stage and could have obscured differences in effect size, measurement, and study context.

Our review included conference abstracts/presentations. However, these sources often provide limited methodological detail and may not have undergone peer review, which may reduce confidence in the completeness and interpretation of the findings. In addition, results reported at the conference may differ from those in later full-text publications.

## Conclusion

The available evidence suggests that the COVID-19 pandemic posed a threat to progress toward eliminating viral hepatitis as a public-health problem by 2030. This scoping review mapped reported changes across the HCV cascade of care. We identified 28 studies, most of which were conducted in Europe and North America. The literature focused mainly on diagnosis and treatment, while linkage to care was the stage most consistently reported as disrupted among the studies that assessed it.

These findings highlight critical gaps in the global evidence base and point to priorities for action. Surveillance and research capacity must be strengthened in high-burden regions so that global data reflect disease patterns and service disruptions in low- and middle-income countries. Routine monitoring of all stages of the cascade of care –not only diagnosis and treatment– is needed to detect service interruptions during future crises. Finally, building resilient HCV care systems is essential. Lessons from HIV programs – such as community-based services, decentralized drug delivery, telemedicine, and strong partnerships with civil society – can guide strategies to maintain continuity of care and sustain progress toward the 2030 viral hepatitis elimination goals.

## Supplementary Information

Below is the link to the electronic supplementary material.

Supplementary Material 1

Supplementary Material 2

Supplementary Material 3

## Data Availability

Not applicable.

## Abbreviations

HCV: Hepatitis C virus
WHO: World Health Organization
UNAIDS: Joint United Nations Programme on HIV/AIDS
SVR: Sustained virologic response
PRISMA-ScR: Preferred Reporting Items for Systematic Reviews and Meta-Analysis extension for Scoping Reviews
PCC: Population, Concept, Context
MeSH: Medical Subject Heading
