# Supplementary material for: Impact of the COVID-19 pandemic on hepatitis C care across the cascade of care: a scoping review
Source: BMC Infect Dis. 2026 Jun 13;26:1139. doi: 10.1186/s12879-026-13799-1 (PMC13264823; doi:10.1186/s12879-026-13799-1)
Supplement: Supplementary file 1 — Supplementary Material 1 [file 12879_2026_13799_MOESM1_ESM.docx]

Additional File 1. Search strategy

| Database | Search Strategy |
| --- | --- |
| PubMed | ((“COVID-19” [Mesh] OR “SARS-CoV-2” [Mesh] OR “COVID-19”[tiab] OR “SARS-CoV-2”[tiab] OR “coronavirus”[tiab] OR “Severe acute respiratory syndrome coronavirus 2”[tiab] OR “2019-nCoV”[tiab] OR “covid”[tiab] OR “pandemic”[tiab] OR “COVID-19 pandemic”[tiab] OR “covid*”[tiab] OR “coronavirus*”[tiab] OR “SARS coronavirus*”[tiab] OR “SARS-COV*” [tiab]) AND (“Hepatitis C” [Mesh] OR “Hepatitis c”[tiab] OR “hcv” [tiab] OR “Hep c” [tiab]) AND (“Health Services” [Mesh] OR “Health Services Research” [Mesh] OR “Delivery of Health Care” [Mesh] OR “Public Health” [Mesh] OR “Facilities and Services Utilization”[Mesh] OR “Ambulatory care”[Mesh] OR “Health services”[tiab] OR “Health services research”[tiab] OR “Delivery of health care”[tiab] OR “Public health”[tiab] OR (“Facilities”[tiab] AND service utilization[tiab]) OR “CoC”[tiab] OR “Cascade of care”[tiab] OR “Care cascade”[tiab] OR “Care continuum”[tiab] OR “Continuity of patient care”[tiab] OR “Treatment”[tiab] OR “Prevention” [tiab] OR “Diagnosis”[tiab] OR “Testing”[tiab] OR “Screening” [tiab] OR “Elimination” [tiab] OR “Care”[tiab] OR “Prescription[tiab] OR “Link”[tiab] OR “Ambulatory care”[tiab] OR “Hospital care”[tiab] OR “Out-patient”[tiab] OR “Outpatient”[tiab])) |
| Web of Science | TS=(“COVID-19” OR “SARS-CoV-2” OR “COVID 19” OR “coronavirus” OR “Severe acute respiratory syndrome coronavirus 2” OR “2019-nCoV” OR “covid” OR “pandemic” OR “COVID-19 pandemic” OR “covid*” OR “coronavirus*” OR “SARS coronavirus*” OR “SARS-COV*”) AND TS=(“Hepatitis C” OR “hcv” OR “Hep c”) AND TS= (“Health Services” OR “Health Services Research” OR “Delivery of Health Care” OR “Public Health” OR (“Facilities” AND “Services Utilization”) OR “Ambulatory care” OR “CoC” OR “Cascade of care” OR “Care cascade” OR “Care continuum” OR “Continuity of patient care” OR “Treatment” OR “Prevention” OR “Diagnosis” OR “Testing” OR “Screening” OR “Elimination” OR “Care” OR “Prescription” OR “Link” OR “Hospital care” OR “Out-patient” OR “Outpatient” |
| Embase | (‘COVID-19’: ti, ab, kw OR ‘SARS-CoV-2’: ti, ab, kw OR ‘Coronavirus’: ti, ab, kw OR ‘Severe acute respiratory syndrome coronavirus 2’: ti, ab, kw 2019-nCoV: ti, ab, kw OR ‘Covid’: ti, ab, kw OR ‘Pandemic’: ti, ab,kw OR ‘COVID-19 pandemic’: ti, ab, kw OR ‘covid*’: ti, ab, kw OR ‘coronavirus*’: ti, ab, kw OR ‘SARS coronavirus*’: ti, ab, kw OR ‘SARS-COV*’: ti, ab, kw) AND (‘Hepatitis C‘: ti, ab, kw OR ‘hcv’: ti, ab, kw OR ‘’Hep c’: ti, ab, kw) AND (‘Health Services’: ti, ab, kw OR ‘Health Services Research’: ti, ab,kw OR ‘Delivery of Health Care’: ti, ab, kw OR ‘Public Health’: ti, ab, kw OR (‘Facilities: ti, ab, kw AND ‘Services Utilization’: ti, ab, kw) OR ‘Ambulatory care’: ti, ab, kw OR ‘CoC’: ti, ab, kw OR ‘Cascade of care’: ti, ab, kw OR ‘Care cascade’: ti, ab, kw OR ‘Care continuum’: ti, ab, kw OR ‘Continuity of patient care’: ti, ab, kw OR ‘Treatment’: ti, ab, kw OR ‘Prevention’: ti, ab, kw OR ‘Diagnosis’: ti, ab, kw OR ‘Testing’: ti, ab, kw OR ‘Screening’: ti, ab, kw OR ‘Elimination’: ti, ab, kw OR ‘Care’: ti, ab, kw OR ‘Prescription’: ti, ab, kw OR ‘Link’: ti, ab, kw OR ‘Hospital care’: ti, ab, kw OR ‘Out-patient’: ti, ab,kw OR ‘Outpatient’: ti, ab, kw) |
| Scopus | (‘COVID-19’: ti, ab, kw OR ‘SARS-CoV-2’: ti, ab, kw OR ‘Coronavirus’: ti, ab, kw OR ‘Severe acute respiratory syndrome coronavirus 2’: ti, ab, kw 2019-nCoV: ti, ab, kw OR ‘Covid’: ti, ab, kw OR ‘Pandemic’: ti, ab,kw OR ‘COVID-19 pandemic’: ti, ab, kw OR ‘covid*’: ti, ab, kw OR ‘coronavirus*’: ti, ab, kw OR ‘SARS coronavirus*’: ti, ab, kw OR ‘SARS-COV*’: ti, ab, kw) AND (‘Hepatitis C‘: ti, ab, kw OR ‘hcv’: ti, ab, kw OR ‘’Hep c’: ti, ab, kw) AND (‘Health Services’: ti, ab, kw OR ‘Health Services Research’: ti, ab,kw OR ‘Delivery of Health Care’: ti, ab, kw OR ‘Public Health’: ti, ab, kw OR (‘Facilities: ti, ab, kw AND ‘Services Utilization’: ti, ab, kw) OR ‘Ambulatory care’: ti, ab, kw OR ‘CoC’: ti, ab, kw OR ‘Cascade of care’: ti, ab, kw OR ‘Care cascade’: ti, ab, kw OR ‘Care continuum’: ti, ab, kw OR ‘Continuity of patient care’: ti, ab, kw OR ‘Treatment’: ti, ab, kw OR ‘Prevention’: ti, ab, kw OR ‘Diagnosis’: ti, ab, kw OR ‘Testing’: ti, ab, kw OR ‘Screening’: ti, ab, kw OR ‘Elimination’: ti, ab, kw OR ‘Care’: ti, ab, kw OR ‘Prescription’: ti, ab, kw OR ‘Link’: ti, ab, kw OR ‘Hospital care’: ti, ab, kw OR ‘Out-patient’: ti, ab,kw OR ‘Outpatient’: ti, ab, kw) |
| Cochrane library bibliographic database | (“COVID-19”(MeSH) OR “SARS-CoV-2”(MeSH) OR (“Coronavirus” OR “Severe acute respiratory syndrome coronavirus 2” OR “2019-nCoV” OR “Covid” OR “Pandemic” OR “COVID-19 pandemic” OR “covid*” “coronavirus*” OR “SARS coronavirus*” OR “SARS-COV*): ti, ab, kw AND (“Hepatitis C”(MeSH) OR (“hcv” OR “Hepatitis c” OR “Hep c”): ti, ab, kw AND (“Health Services”(MeSH) OR “Health Services Research”(MeSH) OR “Delivery of Health Care”(MeSH) OR “Public Health”(MeSH) OR ((“Facilities” AND “Services Utilization”): ti, ab,kw OR “Ambulatory care”(MeSH) OR “Health Services” OR “Health Services Research” OR “Delivery of Health Care” OR “Public Health” OR “Ambulatory care” OR “CoC” OR “Cascade of care” OR “Care continuum” OR “Continuity of patient care” OR “Treatment” OR “Prevention” OR “Diagnosis” OR “Testing” OR “Screening” OR “Elimination” OR “Care” OR “Prescription” OR ”Link” OR “Ambulatory care” OR “Hospital care” OR “Out-patient” OR “Outpatient”): ti, ab, kw |
