# Supplementary material for: Impact of the COVID-19 pandemic on hepatitis C care across the cascade of care: a scoping review
Source: BMC Infect Dis. 2026 Jun 13;26:1139. doi: 10.1186/s12879-026-13799-1 (PMC13264823; doi:10.1186/s12879-026-13799-1)
Supplement: Supplementary file 2 — Supplementary Material 2 [file 12879_2026_13799_MOESM2_ESM.docx]

**Supplementary Text 1. Studies included in the analysis**

1. Yeo YH, Gao X, Wang J, Li Q, Su X, Geng Y, et al. The impact of COVID-19 on the cascade of care of HCV in the US and China. Ann Hepatol. 2022;27(3):100685. doi:https://doi.org/10.1016/j.aohep.2022.100685.
2. Brzdęk M, Dobrowolska K, Pabjan P, Zarębska-Michaluk D. Clinical characteristics and antiviral therapy in patients infected with hepatitis C virus in the interferon‑free era. Pol Arch Intern Med. 2022;132(9) doi:10.20452/pamw.16282
3. Vargas-Accarino E, Martínez-Campreciós J, Domínguez-Hernández R, Rando-Segura A, Riveiro-Barciela M, Rodríguez-Frías F, et al. Cost-effectiveness analysis of an active search to retrieve HCV patients lost to follow-up (RELINK-C strategy) and the impact of COVID-19. J Viral Hepat. 2022;29(7):579-83. doi:https://doi.org/10.1111/jvh.13686
4. Hussain MRA, Hiebert L, Sugiyama A, Ouoba S, Bunthen E, Ko K, et al. Effect of COVID-19 on hepatitis B and C virus countermeasures: Hepatologist responses from nationwide survey in Japan. Hepatol Res. 2022;52(11):899-907. doi:https://doi.org/10.1111/hepr.13819
5. Hartl L, Jachs M, Bauer D, Simbrunner B, Chromy D, Binter T, et al. HCV hotline facilitates Hepatitis C elimination during the COVID-19 pandemic. J Viral Hepat. 2022;29(12):1062-72. doi:https://doi.org/10.1111/jvh.13746
6. Makuza JD, Jeong D, Soe P, Bartlett S, Velásquez García HA, Binka M, et al. Impact of COVID‐19 pandemic on HCV care cascade in Rwanda: Ecological study from July 2019 to June 2021. Clin Liver Dis. 2022;20(1)
7. Kondili LA, Buti M, Riveiro-Barciela M, Maticic M, Negro F, Berg T, et al. Impact of the COVID-19 pandemic on hepatitis B and C elimination: An EASL survey. JHEP Rep. 2022;4(9):100531. doi:10.1016/j.jhepr.2022.100531
8. Cooper MP, Foley H, Damico D, Wright M, Rhudy C, Schadler A, et al. Impact of the COVID-19 pandemic on hepatitis C outcomes at a health-system specialty pharmacy. J Manag Care Spec Pharm. 2022;28(6):667-72. doi:10.18553/jmcp.2022.28.6.667
9. Hoenigl M, Abramovitz D, Flores Ortega RE, Martin NK, Reau N. Sustained Impact of the Coronavirus Disease 2019 Pandemic on Hepatitis C Virus Treatment Initiations in the United States. Clin Infect Dis. 2022;75(1):e955-e61. doi:10.1093/cid/ciac175
10. Gamkrelidze A, Handanagic S, Shadaker S, Turdziladze A, Tsereteli M, Getia V, et al. The impact of COVID-19 pandemic on the 2020 hepatitis C cascade of care in the Republic of Georgia. Public Health. 2022;205:182-6. doi:https://doi.org/10.1016/j.puhe.2022.01.040
11. Jiang SX, Feizi Farivar J, MacIsaac J, Tam E, Choi M, Luyun P, et al. Hepatitis C treatment during the COVID-19 pandemic has similar efficacy with less resource utilization: analysis from the British Columbia HCV network. Hepatology2022. p. S544.
12. Gülen TA, Turunç T, Oruç E, Kaya H, Ünal N. Impact of the COVID-19 Pandemic on the Management of Chronic Hepatitis C Infection: A Cross- Sectional Study. Viral Hepat J. 2023;29(1):30-5. doi:10.4274/vhd.galenos.2023.2023-4-1
13. Musabaev E, Estes C, Sadirova S, Bakieva S, Brigida K, Dunn R, et al. Viral hepatitis elimination challenges in low- and middle-income countries—Uzbekistan Hepatitis Elimination Program (UHEP). Liver Int. 2023;43(4):773-84. doi:https://doi.org/10.1111/liv.15514
14. Hussain MRA, Ali M, Sugiyama A, Hiebert L, Rahman MA, Azam G, et al. The impact of COVID-19 on hepatitis B and C virus prevention, diagnosis, and treatment in Bangladesh compared with Japan and the global perspective. BMC Health Serv Res. 2023;23(1):1137. doi:10.1186/s12913-023-10138-x
15. Shiha G. The impact of COVID-19 pandemic on the elimination program of viral hepatitis in Egypt. 32nd Conference of the Asian Pacific Association for the Study of the Liver (APASL 2023): Hepatology International 2023. p. S45.
16. Mason AR, Radunsky ALeP, Reyes SM, McBryde J, Jain MK. 2502. Best Practice Alert for Hepatitis C Virus Screening Demonstrates Importance of Reminders during Disruptive Times (COVID-19). Open Forum Infect Dis. 2023;10(Supplement_2) doi:10.1093/ofid/ofad500.2120
17. Boyd AB, W; Boesecke, C; Winston, A; Kenyon, C; De Scheerder, MA; Aho, I; Sambatakou, H; Devitt, E; Maltez, F; Llibre, JM; Domingo, P; Reikvam, DH; Mitsura, VM; Szlavik, J; Bakowska, E; Jablonowska, E; Zilmer, K; Cavassini, M; Wandeler, G; Mocroft, A; Peters, L. Disruptions in testing and treatment services for hepatitis C virus during the SARS-CoV-2 epidemic among individuals with HIV susceptible for HCV reinfection: Results from the EuroSIDA study. 19th European AIDS Conference (EACS2023); Poland: HIV MEDICINE; 2023. p. 43-4.
18. Lindqvist K, Wallmofeldt C, Holmén E, Hammarberg A, Kåberg M. Health literacy and changes in pattern of drug use among participants at the Stockholm Needle Exchange Program during the COVID-19 pandemic. Harm Reduction Journal. 2021;18(1):52. doi:10.1186/s12954-021-00499-z
19. Blach S, Blomé M, Duberg A-S, Jerkeman A, Kåberg M, Klasa P-E, et al. Hepatitis C elimination in Sweden: Progress, challenges and opportunities for growth in the time of COVID-19. Liver Int. 2021;41(9):2024-31. doi:https://doi.org/10.1111/liv.14978
20. Laury J, Hiebert L, Ward JW. Impact of COVID-19 Response on Hepatitis Prevention Care and Treatment: Results From Global Survey of Providers and Program Managers. Clin Liver Dis. 2021;17(1):41-6. doi:10.1002/cld.1088
21. Ceccarelli L, Moretti G, Mazzilli S, Petri D, Corazza I, Rizzo C, et al. Evaluating hepatitis C cascade of care surveillance system in Tuscany, Italy, through a population retrospective data-linkage study, 2015–2021. BMC Infect Dis. 2024;24(1):362. doi:10.1186/s12879-024-09241-z
22. Tramonti Fantozzi MP, Ceccarelli L, Petri D, De Vita E, Agostini A, Colombatto P, et al. Hepatitis C epidemiology and treatment outcomes in Italy: Impact of the DAA era and the COVID-19 pandemic. J Viral Hepat. 2024;n/a(n/a) doi:https://doi.org/10.1111/jvh.13983
23. Brouard C, Schwager M, Expert A, Drewniak N, Laporal S, de Lagasnerie G, et al. Impact of Public Policy and COVID-19 Pandemic on Hepatitis C Testing and Treatment in France, 2014–2021. Viruses. 2024;16(5):792.
24. Whitten C, Turner A, Roberts K, Howell B, Sparkes B, Daley P. Population-level cascade of care for hepatitis C in Newfoundland and Labrador. Can Liver J. 2024;7(3):338-44. doi:10.3138/canlivj-2024-0003
25. Kaufman HW, Bull-Otterson L, Meyer WA, Huang X, Doshani M, Thompson WW, et al. Decreases in Hepatitis C Testing and Treatment During the COVID-19 Pandemic. Am J Prev Med. 2021;61(3):369-76. doi:https://doi.org/10.1016/j.amepre.2021.03.011
26. El Sheikh MZ, Young J, Panagiotoglou D, Cooper C, Cox J, Martel-Laferrière V, et al. Progress toward hepatitis C virus elimination among people living with HIV–hepatitis C virus coinfection in Canada. Can Liver J. 2025;8(1):49-62. doi:10.3138/canlivj-2024-0042
27. Basson AA, Weil C, Marx SE, Dylla DE, Collins M, Hadadi S, et al. Road to Hepatitis C Elimination in Israel: Improvements in Linkage to Care (2009-2020). Adv Ther. 2025;42(3):1522-36. doi:10.1007/s12325-024-03102-6
28. Remy A.-J., Roy B., Hervet J., H. B. Lack of COVID-19 impact on managing hepatitis c in prison like the general population! AASLD The liver Meeting: Hepatology 2021. p. 551A.
