# Supplementary material for: Impact of the COVID-19 pandemic on hepatitis C care across the cascade of care: a scoping review
Source: BMC Infect Dis. 2026 Jun 13;26:1139. doi: 10.1186/s12879-026-13799-1 (PMC13264823; doi:10.1186/s12879-026-13799-1)
Supplement: Supplementary file 3 — Supplementary Material 3 [file 12879_2026_13799_MOESM3_ESM.docx]

Additional file 3. Complementary summary table of results

| **Authors** | **Year** | **Country** | **Type of study** | **Study design** | **Population** | **Outcome reported** | **Description of results** |
| --- | --- | --- | --- | --- | --- | --- | --- |
| Lindqvist K, et al. | 2021 | Sweden | Article | Quantitative observational | People diagnosed with HCV | Diagnosis | There was no change in HCV testing between January and October 2020 compared with the corresponding period in 2019 (p=0.26) among people who inject drugs in the Stockholm Needle Exchange Program. |
|  |  |  |  |  |  | Treatment | There was a decrease in HCV treatment initiation between January and October 2020 compared with the corresponding period from January to October 2019 (p<0.05) among people who inject drugs in the Stockholm Needle Exchange Program. |
| Blach S, et al. | 2021 | Sweden | Article | Modelling study | People diagnosed with HCV | Diagnosis | At the national level, the number of people diagnosed with HCV decreased by 27% from January to October 2020 compared with the same period in 2019. |
|  |  |  |  |  |  | Treatment | At the national level, HCV treatment initiation decreased by 55% from January to October 2020 compared with the same period in 2019. |
| Laury J; Hiebert L; Ward JW | 2021 | 44 countries | Article | Quantitative survey | Health providers or managers | Diagnosis | Overall, 90% of participants reported some level of disruption in HCV testing when comparing the month of highest COVID-19 impact with a typical pre-COVID-19 month. |
|  |  |  |  |  |  | Treatment | Overall, 88% of participants reported some level of disruption in treatment provision when comparing the month of highest COVID-19 impact with a typical pre-COVID-19 month. |
| Brzdęk M, et al. | 2022 | Poland | Article | Quantitative observational | People diagnosed with HCV | Linkage to care | The number of patients receiving liver assessment before treatment initiation increased gradually from 176 in 2015-2016 to 271 in 2018. In 2019, the number was 201, and in 2020 it decreased to 113. |
|  |  |  |  |  |  | Treatment | The proportion of treatment-naive patients was 40.9% in 2015-2016 and then increased steadily over consecutive time intervals, reaching 94.0% in 2019. In 2020, there was a slight decrease to 91.2%. |
|  |  |  |  |  |  | Cure | In 2019, 1 of 200 patients (0.5%) did not achieve SVR. In 2020, 3 of 112 patients (2.7%) did not achieve SVR, in addition to 1 death. |
| Vargas-Accarino E, et al. | 2022 | Spain | Article | Quantitative retrospective | People diagnosed with HCV | Diagnosis | During the COVID-19 period (March 2020 to May 2021), there were 1,085 HCV RNA-positive cases, compared with 506 cases during the pre-COVID-19 period (January 2019 to February 2020). |
|  |  |  |  |  |  | Linkage to care | During the COVID-19 period (March 2020 to May 2021), a lower percentage of patients were linked to care compared with the pre-COVID-19 period (January 2019 to February 2020) (27% vs 43%, p<0.0001). |
|  |  |  |  |  |  | Treatment | There was no difference in the percentage of people who started treatment between the two groups (pre-COVID-19: 2.4% vs COVID-19: 2.7%, p=0.723). |
| Hussain MRA, et al. | 2022 | Japan | Article | Quantitative survey | Health providers or managers | Diagnosis | Overall, 51% of participants reported some level of decline in HCV testing when comparing the month of highest COVID-19 impact with a typical pre-COVID-19 month. |
|  |  |  |  |  |  | Treatment | Overall, 45.5% of participants reported no impact of COVID-19 on treatment initiation when comparing the month of highest COVID-19 impact with a typical pre-COVID-19 month. |
|  |  |  |  |  |  | Cure | Overall, 60.7% of participants reported no impact of COVID-19 on cure when comparing the month of highest COVID-19 impact with a typical pre-COVID-19 month. |
| Hartl L, et al. | 2022 | Austria | Article | Quantitative retrospective | People diagnosed with HCV | Treatment | Compared with 2019, treatment initiation declined by 24.0% in 2020 and by 34.1% in 2021. |
|  |  |  |  |  |  | Cure | There was no difference in cure between 2019 and 2020-2021 (84.6% vs 86.0%, respectively; p=0.767). |
| Makuza JD, et al. | 2022 | Rwanda | Article | Ecological | People diagnosed with HCV | Diagnosis | In the pre-COVID-19 period (July 2019 to January 2020), the monthly average number of people tested for HCV was 36,062, which increased to 168,435 during the COVID-19 period (February 2020 to June 2021). Overall, the number of people with a positive anti-HCV test who were tested for HCV RNA also increased. |
|  |  |  |  |  |  | Treatment | Treatment initiation decreased during the first lockdown (March to May 2020) but increased afterward, peaking in September 2020. However, it remained lower in 2021 than in 2019. |
| Kondili LA, et al. | 2022 | 22 countries | Article | Quantitative survey | Health providers or managers | Diagnosis | All participants reported some level of reduction in HCV RNA testing. |
|  |  |  |  |  |  | Linkage to care | New referrals for specialist medical evaluation of chronic HCV decreased by 49% between 2019 and 2020 (p<0.001). |
|  |  |  |  |  |  | Treatment | Overall, 93.5% of participants reported that 51% fewer patients started treatment during the pandemic compared with the previous year (p<0.001). |
| Cooper MP, et al. | 2022 | USA | Article | Quantitative cohort/longitudinal | People diagnosed with HCV | Treatment | The COVID-19 cohort (March 2020 to November 2020) had a lower therapy completion rate than the pre-COVID-19 cohort (January 2018 to March 2020) (89.1% vs 94.6%, respectively; p=0.001). |
|  |  |  |  |  |  | Cure | The COVID-19 cohort (March 2020 to November 2020) had a lower cure rate than the pre-COVID-19 cohort (January 2018 to March 2020) (53.6% vs 65.5%, respectively; p<0.001). |
| Hoenigl M, et al. | 2022 | USA | Article | Quasi-experimental | People diagnosed with HCV | Diagnosis | Monthly anti-HCV testing and HCV RNA testing dropped by >37% during the first months of the COVID-19 pandemic (p<0.001 for both). By the end of 2020, anti-HCV testing had rebounded to pre-COVID-19 levels, whereas HCV RNA testing had increased but remained below pre-COVID-19 levels. |
|  |  |  |  |  |  | Linkage to care | From March to April 2020, the predicted number of HCV genotype tests decreased by 24% (p=0.023). Thereafter, it increased by 0.9% per month (p=0.047). However, by the end of 2020, it remained below pre-COVID-19 levels. |
|  |  |  |  |  |  | Treatment | From March to April 2020, treatment initiation decreased by 31% (p<0.001). It remained low after April 2020 throughout the remainder of 2020. |
| Gamkrelidze A, et al. | 2022 | Georgia | Article | Quantitative retrospective | People diagnosed with HCV | Diagnosis | The number of people tested for HCV decreased by 25.5% in 2020 compared with 2019 (p<0.001). |
|  |  |  |  |  |  | Treatment | In 2020, there was a significantly lower percentage of treatment initiation compared with 2019 (58.7% vs 62.3%, respectively; p<0.001). However, treatment completion was significantly higher in 2020 than in 2019 (84.6% vs 72.9%, respectively; p<0.001). |
|  |  |  |  |  |  | Cure | There was no change in the percentage of cure between 2020 and 2019 (99.2% vs 99.3%, respectively; p=0.64). |
| Gulen T, et al. | 2023 | Turkey | Article | Quantitative cross-sectional | People diagnosed with HCV | Diagnosis | Anti-HCV testing increased from 2018 to 2019 and then decreased in 2020. The trend increased again in January 2021 but decreased again between May and July 2021. Overall, anti-HCV testing remained below pre-COVID-19 levels in 2021. The percentage of HCV RNA tests among people with a positive anti-HCV result was 95.0% in 2018, 99.7% in 2019, 97.7% in 2020, and 90.0% in 2021. |
|  |  |  |  |  |  | Treatment | There was no statistically significant difference in the percentage of people completing treatment between 2018 and 2021 (p=0.087). |
|  |  |  |  |  |  | Cure | Cure was 84.3% in 2019, declined to 63.6% in 2020, and rebounded to 92.7% in 2021. |
| Musabaev E, et al. | 2023 | Uzbekistan | Article | Quantitative cross-sectional | People diagnosed with HCV | Diagnosis | Among people with a positive anti-HCV result, 54% were HCV RNA positive in the pre-COVID-19 period, compared with 53% in the post-COVID-19 period. |
|  |  |  |  |  |  | Linkage to care | Among people with a positive HCV RNA test, 43% had a physician consultation in the pre-COVID-19 period, compared with 34% in the post-COVID-19 period. |
|  |  |  |  |  |  | Treatment | Among people linked to care and with a positive HCV RNA test, 40% were treated in the pre-COVID-19 period, compared with 42% in the post-COVID-19 period. |
| Ceccarelli L, et al. | 2024 | Italy | Article | Quantitative retrospective | People diagnosed with HCV | Diagnosis | Anti-HCV and HCV RNA testing increased in 2016 and 2017. In 2018, anti-HCV and HCV RNA testing decreased. In 2019, both tests showed a slight increase, followed by a decrease in 2020 and a rebound in 2021. |
|  |  |  |  |  |  | Treatment | The number of treatment initiations in 2020 and 2021 was below the expected level for a pre-COVID-19 period. |
| Tramonti Fantozzi MP, et al. | 2024 | Italy | Article | Quantitative retrospective | People diagnosed with HCV | Treatment | Treatment decreased during COVID-19. The COVID-19 pandemic led to the postponement of treatment initiation. |
|  |  |  |  |  |  | Cure | After excluding patients lost to follow-up, SVR12 remained stable across the study periods, with no statistically significant difference between the pre-DAA, post-DAA, and COVID-19 periods (p=0.837). |
| Brouard C, et al. | 2024 | France | Article | Quantitative observational | People diagnosed with HCV | Diagnosis | Between 2014 and 2021, the number of people tested for HCV increased, except in 2020, when it dropped by 8.3%, followed by a rebound in 2021. However, this rebound remained below the expected level for 2021. |
|  |  |  |  |  |  | Treatment | Treatment initiation decreased during the COVID-19 period. The number of patients initiating DAAs fell sharply by 65.5% in April 2020 compared with February 2020 and, despite a subsequent increase, did not return to pre-pandemic levels during 2020-2021. |
| Whitten C, et al. | 2024 | Canada | Article | Quantitative observational | People diagnosed with HCV | Diagnosis | The COVID-19 pandemic was associated with a reduction in HCV testing. |
|  |  |  |  |  |  | Treatment | HCV treatment increased from 19 people in 2017 to 156 people in 2021. However, only 48 people were treated in 2020. |
|  |  |  |  |  |  | Cure | The SVR12 rate increased from 21% in 2017 to 70% in 2021. |
| Hussain MRA, et al. | 2023 | Bangladesh | Article | Quantitative survey | Health providers or managers | Diagnosis | More than 90% of participants reported some level of decline in anti-HCV and HCV RNA testing. |
|  |  |  |  |  |  | Treatment | More than 95% of participants reported some level of decline in treatment initiation. |
| Kaufman HW, et al. | 2021 | USA | Article | Quantitative retrospective | People diagnosed with HCV | Diagnosis | Anti-HCV tests decreased by 59% in April 2020 compared with the corresponding months in 2018 and 2019 and remained 6% below baseline in July 2020. HCV RNA-positive results fell by 62% in March 2020 and remained 39% below baseline by July 2020. |
|  |  |  |  |  |  | Treatment | Treatment decreased by 43% in May 2020, 37% in June 2020, and 38% in July 2020 compared with the corresponding months in 2018 and 2019. |
| Yeo YH, et al. | 2022 | USA and China | Article | Quantitative cross-sectional | People diagnosed with HCV | Treatment | There was no statistically significant difference in treatment completion between the COVID-19 period (January to June 2020) and the pre-COVID-19 period (2019) (p=0.104). |
|  |  |  |  |  |  | Cure | HCV cure-rate assessment decreased by half during the COVID-19 period. |
| Shiha G | 2023 | Egypt | Abstract/conference presentation | Report | People diagnosed with HCV | Diagnosis | There was a 60.7% reduction in HCV RNA testing in 2020 compared with 2019. |
|  |  |  |  |  |  | Linkage to care | New referrals for HCV care decreased by 82.7% in 2020 compared with 2019. |
|  |  |  |  |  |  | Treatment | There was an 82.6% reduction in the number of patients who started treatment during the pandemic period compared with the previous year. |
| Remy AJ, et al. | 2021 | France | Abstract/conference presentation | Quantitative observational | People diagnosed with HCV | Diagnosis | In 2020, anti-HCV and HCV RNA testing did not decrease compared with 2019. |
|  |  |  |  |  |  | Linkage to care | There was no change in liver assessment using FibroScan between 2019 and 2020 among inmates with a positive HCV RNA test. |
|  |  |  |  |  |  | Treatment | There was no change in treatment initiation between 2019 and 2020 among inmates. |
|  |  |  |  |  |  | Cure | Cure decreased from 12 of 12 inmates in 2019 to 3 of 4 inmates in 2020. |
| Mason AR, et al. | 2023 | USA | Abstract/conference presentation | Quantitative retrospective | People diagnosed with HCV | Diagnosis | The hazard ratio for anti-HCV testing increased to 1.31 (95% CI 1.25-1.38), whereas the hazard ratio for HCV RNA testing decreased to 0.79 (95% CI 0.65-0.96) during the COVID-19 period (2020) compared with the pre-COVID-19 period (2018-2019). |
|  |  |  |  |  |  | Linkage to care | There was a lower hazard ratio (0.43, 95% CI 0.28-0.66) of having a clinic visit for specialized medical evaluation during the COVID-19 period compared with the pre-COVID-19 period. |
| Jiang S, et al. | 2022 | Canada | Abstract/conference presentation | Quantitative retrospective | People diagnosed with HCV | Linkage to care | Liver assessment using transient elastography decreased during the COVID-19 period (March 2020 to June 2021) compared with the pre-COVID-19 period (December 2018 to March 2020), from 91% to 52% (p<0.01). |
|  |  |  |  |  |  | Treatment | Treatment initiation declined by 22% during the COVID-19 period, whereas treatment completion increased from 86% to 96% (p<0.01). |
|  |  |  |  |  |  | Cure | Fewer patients obtained HCV RNA tests for SVR during the COVID-19 period than during the pre-COVID-19 period (74% vs 86%, respectively; p<0.01). However, the SVR rate did not differ significantly between groups. |
| Boyd A, et al. | 2023 | Multicountry (number not specified) | Abstract/conference presentation | Quantitative observational | People diagnosed with HCV | Diagnosis | During the first year of COVID-19, there was a decline in HCV testing. |
|  |  |  |  |  |  | Treatment | Treatment initiation declined during COVID-19. |
| El Sheikh MZ, et al. | 2025 | Canada | Article | Quantitative cohort/longitudinal | People diagnosed with HCV | Treatment | Treatment initiation decreased from 32% in 2019 to 24% in 2020 and 15% in 2021. |
|  |  |  |  |  |  | Cure | The annual cure rate increased from 2014 to 2019, then dropped in 2020 and did not recover afterward. |
| Basson AA, et al. | 2025 | Israel | Article | Quantitative retrospective | People diagnosed with HCV | Diagnosis | Compared with 2019, there was a decline of more than 30% in the number of people who received an HCV RNA test for the first time. |
|  |  |  |  |  |  | Linkage to care | Referrals to specialists decreased during the first lockdown of 2020 compared with the month before the onset of COVID-19. |
|  |  |  |  |  |  | Treatment | Compared with 2019, there was a decline of more than 50% in the number of people who purchased HCV treatment for the first time in 2020. |
|  |  |  |  |  |  | Cure | Cure decreased during the first lockdown of 2020 compared with the month before the onset of COVID-19. |
